# Connection Between Chromosomal Location and Function of CtrA Phosphorelay Genes in Alphaproteobacteria

**DOI:** 10.3389/fmicb.2021.662907

**Published:** 2021-04-29

**Authors:** Jürgen Tomasch, Sonja Koppenhöfer, Andrew S. Lang

**Affiliations:** ^1^Department of Molecular Bacteriology, Helmholtz-Center for Infection Research, Braunschweig, Germany; ^2^Department of Biology, Memorial University of Newfoundland, St. John’s, NL, Canada

**Keywords:** CtrA phosphorelay, replication, genome evolution, genome organization, gene expression

## Abstract

Most bacterial chromosomes are circular, with replication starting at one origin (*ori*) and proceeding on both replichores toward the terminus (*ter*). Several studies have shown that the location of genes relative to *ori* and *ter* can have profound effects on regulatory networks and physiological processes. The CtrA phosphorelay is a gene regulatory system conserved in most alphaproteobacteria. It was first discovered in *Caulobacter crescentus* where it controls replication and division into a stalked and a motile cell in coordination with other factors. The locations of the *ctrA* gene and targets of this response regulator on the chromosome affect their expression through replication-induced DNA hemi-methylation and specific positioning along a CtrA activity gradient in the dividing cell, respectively. Here we asked to what extent the location of CtrA regulatory network genes might be conserved in the alphaproteobacteria. We determined the locations of the CtrA phosphorelay and associated genes in closed genomes with unambiguously identifiable *ori* from members of five alphaproteobacterial orders. The location of the phosphorelay genes was the least conserved in the Rhodospirillales followed by the Sphingomonadales. In the Rhizobiales a trend toward certain chromosomal positions could be observed. Compared to the other orders, the CtrA phosphorelay genes were conserved closer to *ori* in the Caulobacterales. In contrast, the genes were highly conserved closer to *ter* in the Rhodobacterales. Our data suggest selection pressure results in differential positioning of CtrA phosphorelay and associated genes in alphaproteobacteria, particularly in the orders Rhodobacterales, Caulobacterales and Rhizobiales that is worth deeper investigation.

## Introduction

Most bacteria possess one circular chromosome. Replication is initiated through unwinding the two DNA strands at the origin of replication (*ori*) and proceeds on both replichores toward the terminus (*ter*). Here, the dimer of newly synthesized chromosomes is resolved, and cell division can be completed (reviewed by Reyes-Lamothe et al., 2012). Close links between replication and organization of genes on the chromosome became evident with the first complete bacterial genomes (Rocha, 2004; Touchon and Rocha, 2016). In many bacteria, genes are preferentially oriented co-directional to replication progression. This pattern probably evolved to avoid collisions between DNA and RNA polymerase complexes (Lang and Merrikh, 2018). In recent years it also became apparent that the specific locations of genes can have a major influence on transcription levels and thereby control physiological processes (Slager and Veening, 2016). For instance, chromosomal location results in differences in the copy-number of genes during replication. Therefore, genes that are more highly expressed, such as those encoding transcription and translation proteins, tend to be conserved near *ori* (Couturier and Rocha, 2006). The importance of gene location has also been validated experimentally: Relocating *Vibrio cholerae* genes encoding ribosomal proteins to the *ter* region resulted in severe growth defects (Soler-Bistué et al., 2015).

Positioning of genes on the chromosome might also be dictated by regulatory needs. In *Escherichia coli* and other gammaproteobacteria, genes coding for nucleoid-associated proteins and regulators are ordered according to their activities during the growth cycle (Sobetzko et al., 2012). For example, *rpoN*, expressed during exponential growth, is located closer to *ori* while *rpoS*, expressed in the stationary phase, is located closer to *ter*. The same trend was found for the targets of these sigma factors. One of the most fascinating examples is how replication-oriented location of regulatory genes is employed to control the timing of *Bacillus subtilis* spore formation (Narula et al., 2015). Here, imbalance between the expression of *ori* and *ter* located members of a phosphorylation chain during replication inhibits activation of the sporulation-inducing transcription factor Spo0A. This ensures that spore formation is only induced in cells with two complete chromosomes.

Proper transcription of the important cell cycle regulatory gene *ctrA* of *Caulobacter crescentus* is dependent on its chromosomal location, too (Reisenauer and Shapiro, 2002). In alphaproteobacteria, the CtrA phosphorelay regulatory system is widely conserved (Brilli et al., 2010; Panis et al., 2015). We recently found that its key regulatory components are concentrated proximal to *ori* and *ter* in the Rhodobacterales *Dinoroseobacter shibae* and *Rhodobacter capsulatus* and, in contrast to *C. crescentus*, the *ctrA* gene itself is located close to *ter* in both organisms (Koppenhöfer et al., 2019).

In this perspective article, we will first provide a brief overview of the CtrA phosphorelay and its role in controlling the cell cycle and other traits in different bacteria. We will focus on how changes in DNA methylation during replication and the formation of a phosphorylation gradient in predivisional cells influence the regulatory system. Then, we will show that chromosomal location of the regulatory genes is conserved to varying degrees within alphaproteobacterial orders and differs among them. We propose that one consequence of the differing gene locations might be altered timing of expression during the cell cycle. Understanding how their positioning shapes the functionality of the CtrA phosphorelay and associated genes might help to explain the evolution of distinct roles in different alphaproteobacterial orders.

## CtrA and Cell Cycle Control in Alphaproteobacteria

**FIGURE 1:**
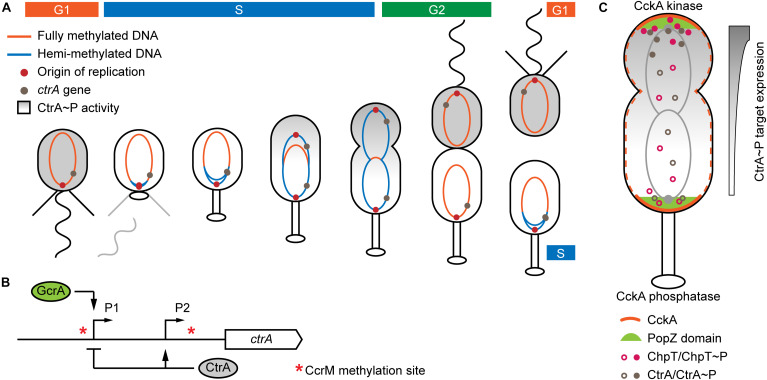
Mechanisms of *C. crescentus* differentiation for which chromosomal localization matters. **(A)** Changes of chromosome methylation state and CtrA activity during the *C. crescentus* cell cycle. Newly replicated DNA stays hemi-methylated during the S phase allowing *ctrA* transcription to be activated. CtrA activity is restricted to the late S phase and the flagellated daughter cell. **(B)** Control of *ctrA* expression. Hemi-methylated P1 is activated by GcrA. Phosphorylated CtrA inhibits expression from P1 and activates expression from P2. **(C)** Establishment of a CtrA activity gradient through localized phosphorylation/dephosphorylation. The protein environment at the new pole triggers kinase functionality of CckA. The PopZ microdomain ensures proximity of phosphorelay components. Panel A inspired by Panis et al. (2015) and panel C inspired by Lasker et al. (2020).

Replicating only once per cell division, the dimorphic bacterium *C. crescentus* displays a eukaryotic-like cell cycle (Figure 1A). The growth-arrested flagellated cell (G1 phase) can transform into a stalked cell that replicates (S phase) and prepares for division (G2 phase) into two physiologically different daughter cells. The old, stalked cell can directly undergo the next round of replication while the new, flagellated cell remains in a growth-arrested state (Degnen and Newton, 1972). Key to the replication-coupled differentiation is the orchestration of gene expression by an array of interconnected regulatory circuits (reviewed by Frandi and Collier, 2019) among which the CtrA phosphorelay takes a leading role (Laub et al., 2000, 2002).

As part of the regulation of cell division events, autophosphorylation of the transmembrane histidine kinase CckA results in phosphorylation of the phosphotransferase ChpT, which in turn phosphorylates the response regulator CtrA (Biondi et al., 2006). Phosphorylated CtrA then activates differentiation-specific genes and inhibits replication initiation by blocking *ori* from binding by the replication initiator DnaA (Quon et al., 1998; Siam and Marczynski, 2000). Replication rounds are controlled via the directed proteolysis of several transcriptional regulators, including fast degradation of CtrA in the stalked cell, mediated by ClpX, in order to enable initiation of replication (reviewed by Jenal, 2009). The chromosomal position influences transcription of *ctrA* and CtrA targets through DNA methylation and establishment of a CtrA phosphorylation gradient, respectively (detailed in the following sections and Figure 1).

The *C. crescentus* chromosome is linearly ordered in the cell with the *ori* located at the stalked or flagellated pole and *ter* oriented at the pole where the division plane will form (Yildirim and Feig, 2018). The CcrM methyltransferase specifically methylates the adenosine in GANTC palindromic motifs. Expression of *ccrM* is restricted to the transition from S to G2 phase (Zweiger et al., 1994; Laub et al., 2000). Thus, newly replicated DNA stays hemi-methylated until replication has finished (Figure 1A). Expression of *ori*-proximal *ctrA* is controlled by two different promoters (Figure 1B). Promoter P1 gets activated in the early S phase and CtrA then triggers its own expression from promoter P2 while inhibiting expression from P1 in pre-divisional cells and the flagellated daughter cell (Domian et al., 1999). Activation of P1 requires hemi-methylation of an upstream GANTC site, and thus the replication fork has to pass the *ctrA* locus for this promoter to be active (Reisenauer and Shapiro, 2002; Mohapatra et al., 2020). Activity of P1 is highest when the respective motif on the coding strand is methylated (Mohapatra et al., 2020). If *ctrA* is moved closer to *ter*, the P1-associated GANTC motif remains in the fully methylated state longer and the resulting delay of *ctrA* transcription leads to elongated flagellated daughter cells. The transcription factor responsible for P1 regulation is GcrA, which is active exclusively in S phase cells and oscillates with CtrA activity (Holtzendorff et al., 2004). GcrA preferentially binds and activates promoters carrying fully or hemi-methylated GANTC motifs (Fioravanti et al., 2013; Haakonsen et al., 2015). Replication-controlled methylation also affects *ftsZ* expression, which encodes the divisome Z-ring protein. In this case the promoter is most active in the fully methylated state (Gonzalez and Collier, 2013). The regulatory function of CcrM is probably conserved broadly in alphaproteobacteria as GANTC motifs are enriched in intergenic regions on the vast majority of chromosomes (Gonzalez et al., 2014).

CckA is dispersed throughout the inner membrane, but concentrates at the cell poles in pre-divisional cells (Angelastro et al., 2010). It acts as a kinase at the new cell pole and as a phosphatase elsewhere. The switch in enzymatic activity is controlled by interaction with different sets of proteins (Tsokos et al., 2011). Essential for triggering the kinase activity of CckA are its homo-oligomerization and direct interaction with the pseudo-kinase DivL, both concentrated at the cell poles (Mann and Shapiro, 2018). Recently, Lasker et al. (2020) demonstrated the formation of diffusion-limiting microdomains at the cell poles that ensure close proximity of CckA, ChpT and CtrA in order to allow efficient phosphotransfer (Figure 1C). The polar localization of phosphorylating and dephosphorylating enzymatic chains ensures the formation of a CtrA activity gradient from the flagellated to the stalked pole in pre-divisional cells. When a promoter that is regulated exclusively by CtrA was repositioned on the chromosome, its expression decreased along the *ori-ter* axis, in accordance with the increasing distance from the flagellated cell pole (Lasker et al., 2020).

The core components of the CtrA phosphorelay are highly conserved within the alphaproteobacteria and connected to accessory regulatory systems that are often restricted to specific orders (Brilli et al., 2010). In particular, most genes of the polarity module (van Teeseling and Thanbichler, 2020) essential for dimorphic development of *C. crescentus* are found only in members of the Caulobacterales and Rhizobiales orders, an exception being the more widely conserved *divL* gene. The CtrA regulon also differs among orders (Brilli et al., 2010; Panis et al., 2015). Flagellar genes are controlled by CtrA in all studied orders, including the early branching Rhodospirillales, leading to the hypothesis that regulation of motility was the primordial role of CtrA and cell cycle control was acquired later (Greene et al., 2012). Transcriptional activation of the DNA repair machinery, observed in several species, might also be a more ancient function of CtrA (Poncin et al., 2018).

Construction of *ctrA* knockout mutants failed or they showed severe growth defects in the Caulobacterales *C. crescentus* (Quon et al., 1996; Christen et al., 2011; Guzzo et al., 2020) and *Hyphomonas neptunium* (Leicht et al., 2020) as well as the Rhizobiales *Sinorhizobium meliloti* (Barnett et al., 2001) and *Brucella abortus* (Bellefontaine et al., 2002), but has no negative effects on growth or viability in the Sphingomonadales *Sphingomonas melonis* (Francez-Charlot et al., 2015), Rhodospirillales *Rhodospirillum centenum* (Bird and MacKrell, 2011) and *Magnetospirillum magneticum* (Greene et al., 2012), as well as the Rhodobacterales members studied so far (Miller and Belas, 2006; Mercer et al., 2010; Zan et al., 2013; Wang et al., 2014; Hernández-Valle et al., 2020). While the influence of CtrA on replication has been demonstrated in *Sphingomonas melonis* and some Rhodobacterales, these bacteria lack a strict dimorphic lifestyle or polar growth that has been demonstrated for the Caulobacterales and Rhizobiales. These findings led to the hypothesis that essentiality of *ctrA* arose during the evolution of a lifestyle that couples differentiation to reproduction (van Teeseling and Thanbichler, 2020). An intermediate step might have been a non-essential influence on replication and cell division.

## Conserved Location of CtrA-Associated Genes in Alphaproteobacterial Orders

Given that chromosomal localization influences expression of cell cycle-controlling genes in the model alphaproteobacterium *C. crescentus* and knowing that genes key to this process are conserved within this class, we asked if the location of these genes shows a pattern and if there are differences among orders in which the CtrA phosphorelay is an essential regulator of replication-coupled differentiation and those in which it is not. We used a dataset of 179 closed genomes from five alphaproteobacterial orders for which Ori-Finder (Gao and Zhang, 2008) unambiguously identified *ori* and identified the orthologs of CtrA phosphorelay and associated genes using Proteinortho (Lechner et al., 2011). Only one representative strain was selected for each species to avoid species overrepresentation bias. Detailed analysis steps can be found in the Supplementary Material. The analyzed genomes from the Rhodospirillales, Sphingomonadales, Rhodobacterales, Caulobacterales and Rhizobiales are listed in Supplementary Table 1, and the analyzed genes are listed in Supplementary Tables 2, 3.

**FIGURE 2:**
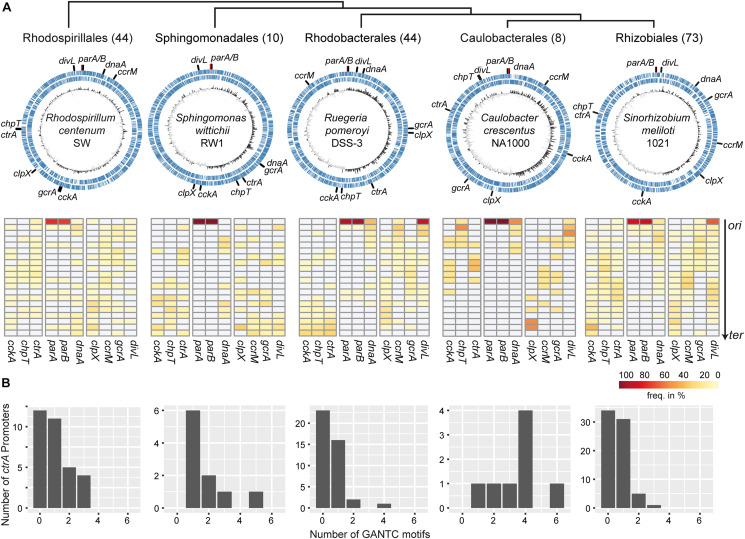
Chromosomal localization of CtrA phosphorelay component genes and methylation of the *ctrA* promoter region in alphaproteobacteria. **(A)** Alphaproteobacterial orders are arranged according to Muñoz-Gómez et al. (2019). The numbers of genomes per order are in brackets. Upper panel: representative chromosomes for each order with positions of regulators marked. Lower panel: percentage of regulator genes within 20 segments of the chromosomes of the particular order oriented along the *ori-ter* axis. **(B)** Distribution of the number of CcrM methylation motifs in the *ctrA* promoter regions for each order.

Figure 2A summarizes the localization analysis with the upper panel showing one representative genome and the lower panel showing the frequency of the respective genes within 20 segments on the *ori-ter* axis. The orders are arranged phylogenetically with the earliest branching Rhodospirillales at the left and the latest branching Rhizobiales on the right (Muñoz-Gómez et al., 2019). In almost all analyzed genomes the *parAB* genes were located close to *ori* with some exceptions in the Rhodospirillales, Rhodobacterales, and Rhizobiales. This is in accordance with previous studies that found the *par* locus predominantly conserved close to *ori* (Livny et al., 2007). The location of the other analyzed genes and the respective conservation differed among the orders.

We observed no conserved localization of the genes examined in the Rhodospirillales with the exceptions of *parAB* and *clpX*, which tended to be located closer to *ter* (Figure 2A). Surprisingly, and in contrast to the other orders, *ctrA*, *gcrA* and *ccrM* were identified in 72–82% of the genomes, whereas *chpT*, *cckA* and *divL* were identified in only 27–36% (Supplementary Table 1). This might indicate that the selection pressure to maintain these genes is lower in Rhodospirillales than in the other orders. However, CckA and DivL are modular proteins, therefore their architecture might have evolved differently in this order and ChpT is a small protein that also shows greater divergence within orders (Brilli et al., 2010; Mercer et al., 2012), making definitive identification of homologs more difficult. Similarly, no clear distribution patterns of the genes analyzed were observed in the Sphingomonadales genomes. The only exceptions were *cckA* and *chpT*, which tended to be localized near *ter* (Figure 2A). Of note, in the Rhodospirillales and Sphingomonadales, the *dnaA* gene was not conserved close to *ori*, in contrast to the other three orders.

In the Caulobacterales the phosphorelay genes *ctrA*, *cckA* and *chpT* as well as *divL* showed conserved localization in the half of the chromosome closer to *ori*. The *clpX* gene was highly conserved in proximity to *ter*. By contrast, *ccrM* and *gcrA* were predominantly found midway between *ori* and *ter*. In the Rhizobiales, localization of *cckA* was conserved in the “lower half” of the chromosome with a peak close to *ter* while *chpT* and *ctrA* were preferentially located midway between *ori* and *ter*. The genes *clpX*, *ccrM* and *gcrA* showed similar trends as in the Caulobacterales. Interestingly, in many of the Rhizobiales genomes we identified two *divL* homologs, one of which was conserved near *ori* (Supplementary Table 2).

In the Rhodobacterales a clear preference for *ter*-proximal localization was observed for *cckA*, *chpT* and *ctrA*, while *ccrM* was preferentially located close to *ori*. Like in the Rhizobiales, several genomes contained two paralogs of *divL* that were located mostly near *ori* (Supplementary Table 2). We also analyzed the chromosomal position of other genes that are part of the CtrA regulon in this order and that regulate the gene transfer agent (GTA) gene cluster (Supplementary Table 3). The direct activator of the GTA cluster *gafA* (Fogg, 2019) and its neighbor (Dshi_1585 in *D. shibae*) were located in proximity to *ter* while the *rbaVW* genes that encode part of a partner-switching phosphorelay system (Mercer and Lang, 2014) were preferentially found close to *ori* (Supplementary Figure 1). Interestingly, the CtrA-controlled genes (Koppenhöfer et al., 2019) that are part of the DNA uptake and recombination machinery (*lexA*, *recA*, and *comECFM*) also showed a conserved location pattern.

As (hemi)-methylation is an important factor in the regulation of *ctrA* expression in *C. crescentus* we determined the number of GANTC motifs 300 bp upstream of the *ctrA* homologs in all orders (Figure 2B). All putative *ctrA* promotors contained at least one and up to five or six CcrM methylation sites in the Sphingomonadales and Caulobacterales, respectively. In 65% and 50% of all putative Rhodospirillales and Rhizobiales *ctrA* promoters, respectively, we identified the GANTC motif. The lowest number was found in Rhodobacterales where only 45% of all promoter regions contained this motif.

## Discussion

Here, we evaluated whether or not key regulators associated with the CtrA phosphorelay have conserved chromosomal locations. The number of genomes available to analyze was small for the orders Caulobacterales and Sphingomonadales, leaving the possibility of a bias in our study. The employed Ori-Finder tool returned several possible *ori* positions for a considerable number of genomes that we excluded from further analysis. We found that the *parAB* genes might serve as a good anchor for manual curation of the *ori* position. The locations of *ori* and *ter* can also be identified experimentally by sequencing DNA from growing cultures when there will be a coverage gradient decreasing from start toward end of replication (Skovgaard et al., 2011; Jung et al., 2019). This could be considered for all future genome sequencing projects.

Despite the limitations, we could identify localization patterns in all orders except for the early branching Rhodospirillales in which the conservation of the CtrA phosphorelay was also lower than in the other orders. Particularly striking was the strong conservation of the phosphorelay genes near *ter* on the Rhodobacterales chromosomes. This conserved localization is also remarkable because core genes in this order show very distinct location patterns among different species (Kopejtka et al., 2019). Localization near *ter* and the low occurrence of GANTC motifs in the *ctrA* promoter might indicate that replication-mediated changes of the state of DNA methylation do not play a major role in regulation of gene expression in this order. On the other hand, establishment of a CtrA phosphorylation gradient might indeed also play a role in Rhodobacterales. The bifunctionality of CckA as a kinase and phosphatase has recently been demonstrated for *R. capsulatus* (Farrera-Calderon et al., 2020).

In some Rhodobacterales the CtrA phosphorelay is integrated into quorum sensing (QS) regulation (Leung et al., 2013; Zan et al., 2013; Wang et al., 2014). CtrA-mediated QS communication induces subpopulation-specific responses, most notably the “decision” of a small number of cells to produce GTAs (Ding et al., 2019; Koppenhöfer et al., 2019). A loss of the CtrA phosphorelay genes is not lethal, the bacteria just resemble a “silent” population. The location of the phosphorelay genes close to *ter* might indicate that communication-induced differentiation is uncoupled from replication and cell division. It is also tempting to speculate that the location of genes controlling GTA expression at the opposite poles of the chromosome ensures repression of GTA production during replication, similar to spore formation in *B. subtilis* (Narula et al., 2015). Indeed, no DNA packaging bias along the *ori-ter* axis has been observed for GTAs, which would be expected if they are produced in replicating cells (Hynes et al., 2012; Tomasch et al., 2018). In the Rhizobiales and Caulobacterales, however, most of the essential CtrA phosphorelay genes are located toward the upper half of the chromosome. This might result in their activation during replication as observed for *ctrA* of *C. crescentus* (Reisenauer and Shapiro, 2002), leading to an interconnected essentiality of reproduction and physiological differentiation. Most essential *C. crescentus* genes are concentrated near *ori* or *ter* (Christen et al., 2011). It would be interesting to see if this pattern is conserved in other species with a pronounced dimorphic lifestyle.

In conclusion, our analysis suggests selection pressure to fix the position of CtrA phosphorelay and associated genes in different chromosomal regions depending on their involvement in different cell physiological processes. This is particularly evident in the Rhodobacterales, Caulobacterales and Rhizobiales. Understanding the underlying evolutionary forces will require both comparative genomic analysis and experimental data beyond what is currently available for a limited number of established model organisms. Our analysis concentrated on the core components of the CtrA phosphorelay but could be expanded to include more accessory regulators and CtrA targets in the different orders. It would also be interesting to identify highly related strains where recent chromosome rearrangements have led to different positions of genes of interest. In *Pseudomonas aeruginosa* a large-scale chromosome inversion resulted in large gene expression and physiological differences between two strains (Irvine et al., 2019). Similarly, analyzing the consequences of relocating genes, as has been done for *C. crescentus* and several other organisms, is a promising experimental approach for understanding the effects of chromosome positioning on gene regulation (Reisenauer and Shapiro, 2002; Gonzalez and Collier, 2013; Soler-Bistué et al., 2015; Lasker et al., 2020).

## Data Availability Statement

The original contributions presented in the study are included in the article/Supplementary Material, further inquiries can be directed to the corresponding authors.

## Author Contributions

JT designed the study. SK analyzed the data. JT and SK visualized the data. JT, SK, and ASL wrote the manuscript. All authors contributed to the article and approved the submitted version.

## Conflict of Interest

The authors declare that the research was conducted in the absence of any commercial or financial relationships that could be construed as a potential conflict of interest.
