# Supplementary material for: Connection Between Chromosomal Location and Function of CtrA Phosphorelay Genes in Alphaproteobacteria
Source: Front Microbiol. 2021 Apr 29;12:662907. doi: 10.3389/fmicb.2021.662907 (PMC8116508; doi:10.3389/fmicb.2021.662907)
Supplement: Supplementary file 1 [file Data_Sheet_1.PDF]

Supplementary Material

Perspective

Connection between chromosomal location and function of CtrA phosphorelay genes in Alphaproteobacteria

Jürgen Tomasch[1#*], Sonja Koppenhöfer[2#], Andrew S. Lang[2*]

[1]Department of Molecular Bacteriology, Helmholtz-Center for Infection Research, Braunschweig, Germany

[2]Department of Biology, Memorial University of Newfoundland, St. John's, Canada

#equally contributing authors

*Correspondence: juergen.tomasch@helmholtz-hzi.de, aslang@mun.ca

Key words: CtrA phosphorelay, replication, genome order, genome evolution, gene regulation

This file contains:

Supplementary Methods

Supplementary Figure S1

Legends for Supplementary Tables S1-S3

Supplementary Methods

Genome dataset and identification of the origin of replication

Data for closed genomes of five alphaproteobacterial orders, the Rhodospirillales, Sphingomondales, Rhodobacterales, Caulobacterales and Rhizobiales were obtained from the NCBI genome assembly database https://www.ncbi.nlm.nih.gov/assembly. Downloaded data formats included fasta nucleic acid (fna), fasta protein (faa), genbank (gbff) and genomic featuere format (gff). The largest replicon was assumed to be the (major) chromosome and used for subsequent analyses. Accession numbers of genomes used are provided in the supplementary table 1. The origin of replication (ori) was located using Ori-Finder (Gao and Zhang, 2008) and default settings. Ori-finder incorporates information on base-disparity, DnaA-boxes and, in case annotation is provided, also the positions of genes frequently found near *ori*. We chose the default DnaA-box TTATCCACA allowing for one mismatch. The required ptt files for gene similarity search were generated (https://github.com/sgivan/gb2ptt#gb2ptt) from gbff files. All ori-finder results were manually checked. Only chromosomes where one *ori* was unambiguously identified, were subsequently included in the investigation. The full ori-finder output is available from the authors upon request.

Gene position was recalculated in relation to ori using custom R scripts (R version 4.0.3). For each chromosome, the gene positions were separated into 20 bins based on the relative position to ori. The GC skew was calculated as (G-C)/(G+C) for a sliding window of 10000 bp based on the fna file of the respective genome.

Ortholog identification

To identify homologous proteins and thus gene families in the genomes from the different orders, an ortholog matrix was generated for each order using Proteinortho version v5.16b

(Lechner et al., 2011). The criteria to be considered a homolog were an e-value of 1e-05, identity of 30% and coverage of 75%. Initial identification of CtrA-regulon genes were based on *C. vibrioides* and *D. shibae*. Based on the identification of genes of interest in one organism, this ortholog matrix was then used to identify homologs in the other genomes. The reference organisms are *Dinoroseobacter shibae* DSM16493, *Magnetospirillum magneticum* AMB-1, *Sphingopyxis alaskensis* RB2256, *Brevundimonas subvibrioides* ATCC 15264 and *Brucella suis* 1330 for the Rhodobacterales, Rhodospirillales, Sphingomondales, Caulobacterales and Rhizobiales, respectively.

Visualization

Circular genome plots were generated with the R package ggbio (1.38.0). The sum of binned distances to ori for each gene family were combined into a matrix and data for each gene normalized to the sum of its occurrence and visualized with the R package pheatmap (1.0.12).

Identification of CtrA-promoter methylation

300 bp upstream of the *ctrA* start codon were searched for the GANTC motif using the function matchPattern from the Biostrings package (2.58.0). Binding site distribution was visualized with ggplot2 (3.3.3).

References

Gao, F., and Zhang, C. T. (2008). Ori-Finder: A web-based system for finding oriCs in unannotated bacterial genomes. *BMC Bioinformatics* 9. doi:10.1186/1471-2105-9-79.

Lechner, M., Findeiß, S., Steiner, L., Marz, M., Stadler, P. F., and Prohaska, S. J. (2011). Proteinortho: Detection of (Co-)orthologs in large-scale analysis. *BMC Bioinformatics* 12. doi:10.1186/1471-2105-12-124.

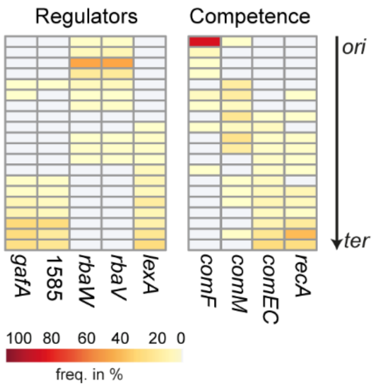

Supplementary Figure S1. Chromosomal localization of Rhodobacterales genes coding for regulators of GTA gene expression and the competence machinery involved in DNA uptake from GTA particles. Visualization is based on data found in Supplementary Table S3.

Supplementary Tables

Supplementary Table S1. CtrA-core gene distribution among all analyzed species. Accession number of the genbank assembly (https://www.ncbi.nlm.nih.gov/assembly) is shown.

Supplementary Table S2. Identified CtrA core genes of all orders. Accession = genbank assembly; Replicon = Genbank ID of the respective chromosome; OStart = gene start recalculated relative to ori; PStart = OStart as percentage of chromosome size.

Supplementary Table S3. Identified Rhodobacterales CtrA regulon genes involved in GTA production and recombination. Accession = genbank assembly; Replicon = Genbank ID of the respective chromosome; OStart = gene start recalculated relative to ori; PStart = OStart as percentage of chromosome size.
